# IL-40: A New B Cell-Associated Cytokine Up-Regulated in Rheumatoid Arthritis Decreases Following the Rituximab Therapy and Correlates With Disease Activity, Autoantibodies, and NETosis

**DOI:** 10.3389/fimmu.2021.745523

**Published:** 2021-10-21

**Authors:** Adela Navrátilová, Lucie Andrés Cerezo, Hana Hulejová, Viktor Bečvář, Michal Tomčík, Martin Komarc, David Veigl, Dana Tegzová, Jakub Závada, Marta Olejárová, Karel Pavelka, Jiří Vencovský, Ladislav Šenolt

**Affiliations:** ^1^ Department of Experimental Rheumatology, Institute of Rheumatology, Prague, Czechia; ^2^ Department of Rheumatology, First Faculty of Medicine, Charles University, Prague, Czechia; ^3^ Department of Methodology, Faculty of Physical Education and Sport, Charles University, Prague, Czechia; ^4^ First Orthopaedic Clinic, 1^st^ Faculty of Medicine, Charles University, Prague, Czechia

**Keywords:** interleukin-40, rheumatoid arthritis, autoantibodies, disease activity, B cells, NETosis

## Abstract

**Background:**

Interleukin 40 (IL-40) is a newly identified B cell-associated cytokine implicated in humoral immune responses and B cell homeostasis. As B cells play a pivotal role in autoimmunity, we investigated the function of IL-40 in rheumatoid arthritis (RA).

**Methods:**

IL-40 expression was determined in the synovial tissue from RA and osteoarthritis (OA) patients. IL-40 was analysed in the serum/synovial fluid of patients with RA (n=50), systemic lupus erythematosus (SLE, n=69), OA (n=44), and healthy controls (HC, n=50). We assessed the changes of IL-40 levels in RA patients following the B cell depletion by rituximab (n=29) or after the TNF inhibition by adalimumab (n=25). We examined the relationship between IL-40, disease activity, autoantibodies, cytokines, and NETosis markers. Effect of IL-40 on synovial fibroblasts was determined.

**Results:**

IL-40 was overexpressed in RA synovial tissue, particularly by synovial lining and infiltrating immune cells. The levels of IL-40 were up-regulated in the synovial fluid of RA versus OA patients (p<0.0001). Similarly, IL-40 was increased in the serum of RA patients compared to HC, OA, or SLE (p<0.0001 for all) and decreased after 16 and 24 weeks (p<0.01 and p<0.01) following rituximab treatment. No significant effect of adalimumab on IL-40 was observed. IL-40 levels in RA patients correlated with rheumatoid factor-IgM and anti-cyclic citrullinated peptides (anti-CCP) in the serum (p<0.0001 and p<0.01), as well as in the synovial fluid (p<0.0001 and p<0.001). Synovial fluid IL-40 was also associated with disease activity score DAS28 (p<0.05), synovial fluid leukocyte count (p<0.01), neutrophil attractants IL-8 (p<0.01), MIP-1α (p<0.01), and markers of neutrophil extracellular traps externalization (NETosis) such as proteinase 3 (p<0.0001) and neutrophil elastase (p<0.0001). Synovial fibroblasts exposed to IL-40 increased the secretion of IL-8 (p<0.01), MCP-1 (p<0.05), and MMP-13 (p<0.01) compared to the unstimulated cells.

**Conclusions:**

We show the up-regulation of IL-40 in RA and its decrease following B cell depleting therapy. The association of IL-40 with autoantibodies, chemokines, and markers of NETosis may imply its potential involvement in RA development. Moreover, IL-40 up-regulates the secretion of chemokines and MMP-13 in synovial fibroblasts, indicating its role in the regulation of inflammation and tissue destruction in RA.

## Introduction

Rheumatoid arthritis (RA) is a common chronic autoimmune disease characterized by persistent synovitis with various extra-articular manifestations (1). The pathogenic process of RA is localised particularly in the synovial joint infiltrated by immune cells, which together with synovial fibroblasts release inflammatory mediators and matrix-degrading enzymes, contributing to bone erosion and cartilage destruction (2). For decades, RA has been widely accepted as a T cell-driven autoimmune disease. Emerging evidence emphasized the significance of B cells in the pathogenesis of RA beyond antibody production (3). As documented in the experimental models of arthritis, cytokines secreted by B cells may be involved in the induction and promotion of arthritis (4, 5). Recently, B cell depleting agents have proved highly effective in RA treatment (6). Nevertheless, elimination of B cells often results in a decline of autoantibody levels in RA (7), but does not always correlate with the clinical response to therapy (7, 8). It is apparent that autoantibody-independent mechanisms of B cells are implicated in the progression of RA (9). Therefore, identification of new B cell-associated biomarkers embedded in the pathogenesis of RA would be beneficial.

Interleukin 40 (IL-40) is recently identified as a B cell-associated cytokine, which is related to immune response mechanisms and B cell homeostasis (10). This cytokine was originally described by Catalan-Dibene et al. in 2017 as a small (27 kDa) secreted protein encoded by the *C17orf99* gene (chromosome 17 open reading frame 99) (10). Based on its unique structural properties, IL-40 fell into the category of the few so-called “orphan” cytokines (11), which do not share homologies with any of the established cytokine families. To date, there have been only few studies regarding the *C17orf99* gene or its product IL-40 protein. It is well established that C17orf99 or IL-40 are expressed only in mammals and are particularly enhanced in fetal liver, bone marrow, and activated B cells (10–13). As demonstrated in IL-40 knockout mice, IL-40 is associated with lactation and affects the production of IgA and, thereby, the composition of the intestinal microbiome in mice (10). Moreover, IL-40 knockout mice exhibited abnormalities in B cell populations, indicating the role of IL-40 in B cell development (10). Under *in vitro* conditions, IL-40 is expressed by human B cells upon activation by anti-CD40 mAb, anti-IgM, and IL-4, and its expression is further potentiated by transforming growth factor (TGF)-β1 (10). Furthermore, IL-40 was detected in several cell lines of human diffuse large B cell lymphoma (10), and its differential expression among the lymphoma subtypes was reported (14, 15). Very recently, C17orf99 was found down-regulated in the co-culture of human respiratory epithelial cells with macrophages upon treatment with anti-inflammatory cytokine IL-38 (16), suggesting the role of IL-40 in inflammation. The only evidence linking IL-40 to autoimmune inflammation was reported back in 2012 by Zingaretti et al., who identified *C17orf99* as one of the four autoantigens discriminating autoimmune hepatitis from healthy individuals (17).

Altogether, the implication of IL-40 in the B cell homeostasis and in the regulation of immune mechanisms makes it a suitable candidate player in the pathogenesis of autoimmune diseases. Thus, we aimed to analyse the expression of IL-40 in patients with RA, its association with disease-specific parameters, and its immunomodulatory capacity *in vitro*.

## Materials and Methods

### Patients

This study included three cohorts of patients with RA. Cohort 1 involved patients with active RA with knee effusion (total of 50 patients (31 women and 19 men) with a mean (SD) age of 55 (13) years and a disease duration of 14 (12) years. A total of 68% RA patients were IgM rheumatoid factor (RF-IgM) positive and 58% anti-CCP positive. Cohort 2 included patients with RA indicated for the treatment with rituximab (MabThera, Roche): a total of 29 patients (26 women and 3 men) with a mean (SD) age of 49 (14) years and a disease duration of 14 (10) years. In cohort 2, 72% of patients were IgM rheumatoid factor (RF-IgM) positive, 69% anti-CCP positive, and 82% of patients received anti-Tumor necrosis factor (TNF) therapy prior to the administration of rituximab. Rituximab, a chimeric monoclonal anti‐human CD20 antibody, which primarily causes B cell depletion, was administrated as a 1000 mg intravenous infusion at baseline and on day 14. Cohort 3 involved patients with RA indicated for the treatment with the TNF inhibitor (TNFi) adalimumab (Humira, Abbott Laboratories): a total of 25 patients (22 women and 3 men) with a mean (SD) age of 50 (13) years and a disease duration of 7 (7) years. A total of 68% RA patients were RF-IgM positive and 72% anti-CCP positive. Adalimumab was administrated subcutaneously at a standard dose of 40 mg every other week. In all patients, RA was diagnosed according to the 2010 American College of Rheumatology (ACR)/European League Against Rheumatism (EULAR) criteria (18). The control groups comprised of 44 patients with knee OA (33 women and 11 men) with a mean (SD) age of 67 (12) years and a disease duration of 7 (6) years, and 50 healthy controls (HC) (37 women and 13 men) with a mean (SD) age of 52 (14) years. Demographic and clinical characteristics of the study subjects are shown in **Table 1**. In addition, a cohort of 69 patients with systemic lupus erythematosus (SLE) (62 women and 7 men) with a mean (SD) age of 43 (16) years and a disease duration of 2 (8) years, fulfilling the 2012 Systemic Lupus International Collaborating Clinics Classification Criteria (19), was included in the study as it represents an autoimmune rheumatic disease where B cells carry out a central role in the pathogenesis. Written informed consent was obtained from all patients and healthy individuals participating in the study. This study was approved by the Ethics Board of the Institute of Rheumatology in Prague.

**Table 1:** Characteristics of patients with rheumatoid arthritis (RA), osteoarthritis (OA), systemic lupus erythematosus (SLE) and healthy controls (HC).

Characteristics	RA			OA	SLE	HC
	Cohort 1 (Knee joint effusion)	Cohort 2 (RTX therapy)	Cohort 3 (TNFi therapy)			
**Number of patients**	50	29	25	44	69	50
**Gender (F/M)**	31/19	26/3	22/3	33/11	62/7	37/13
**Age (years)**	54.0 (46.0-65.0)	50.0 (39.5-58.0)	52.0 (40.5-59.5)	67.0 (58.0-77.0)	43.0 (29.0-55.0)	55.0 (41.5-62.5)
**Disease duration (years)**	10.0 (6.0-19.0)	13.2 (5.7-23.1)	5.0 (3.0-11.5)	6.5 (2.0-10.8)	1.0 (0.0-2.0)	–
**CRP (mg/l)**	17.5 (5.2-39.5)	23.7 (8.4-31.6)	13.1 (7.6-39.4)	2.5 (1.4-5.4)	1.9 (0.9-22.3)	1.3 (0.6-2.5)
**ESR (mm/h)**	29.5 (17.8-53.0)	50.0 (27.5-68.5)	33.0 (20.0-62.5)	9.5 (6.0-19.2)	–	–
**DAS28 score (ESR)**	5.3 (4.1-6.5)	6.5 (5.7-7.5)	6.4 (5.8-7.0)	–	–	–
**RF positivity, n (%)**	34 (68%)	21 (72%)	17 (68%)	–	–	–
**anti-CCP positivity, n (%)**	29 (58%)	20 (69%)	18 (72%)	–	–	–
**Current medication(csDMARDs/GCs)**	36/26	22/26	25/12	–	–	–
**bDMARDs**	20*	29**	25	–	–	–

Anti-CCP, anti-cyclic citrullinated peptide antibody; CRP, C-reactive protein; DAS28 score, disease activity score; csDMARDs, conventional synthetic disease-modifying antirheumatic drugs; bDMARDs, biological disease-modifying antirheumatic drugs; ESR, erythrocyte sedimentation rate; F, female; GCs, glucocorticoids; HC, healthy controls; M, male; OA, osteoarthritis; RA, rheumatoid arthritis; RF, rheumatoid factor; RTX, rituximab; SLE, systemic lupus erythematosus; TNFi, tumour necrosis factor inhibitor. Data are expressed as median (IQR).

*Out of 20 patients, 15 were treated with anti-TNF therapy, 4 with anti-CD20 therapy, and 1 with anti-IL-6 therapy. **Out of 29 patients treated with rituximab, 24 received anti-TNF therapy prior to the administration of rituximab.

### Laboratory Measurements

In cohort 1, paired blood and synovial fluid samples were collected at the time of clinically indicated knee arthrocentesis in 50 RA and 44 knee OA patients. In cohorts 2 and 3, only peripheral blood samples were obtained from 29 RA patients who received rituximab therapy (cohort 2) at baseline and at weeks 16 and 24 and from 25 patients receiving adalimumab (cohort 3) at baseline and at weeks 12 and 52. All samples were immediately processed, aliquoted, and stored at -80°C until use. Prior to analysis, the synovial fluid samples were treated with hyaluronidase (Hylase Dessau; Riemser Arzneimittel, Greifswald, Germany) for 30 minutes at 37°C. The disease severity was assessed by the disease activity score in 28 joints using the erythrocyte sedimentation rate (DAS28-ESR). Levels of C-reactive protein (CRP) were analysed by turbidimetry using an Olympus Biochemical Analyzer (Olympus CO Ltd., Tokyo, Japan), and anti-CCP and RF-IgM levels were determined using standard enzyme-linked immune sorbent assay (ELISA) kits (Test Line s.r.o., Czech Republic). CD19+ B cells were assessed by flow cytometry as previously described (20).

### Measurement of Cytokines

Levels of IL-40 in the serum and synovial fluid were analysed by commercially available ELISA kits, according to the manufacturer’s protocol (MyBioSource, San Diego, USA). The detection limit of the assay is 0.1 ng/ml. The levels of neutrophil extracellular traps externalization process (NETosis) associated markers proteinase 3 (PR3) and neutrophil elastase (NE) were analysed by commercially available kits (Biovendor, Brno, Czech Republic, and Abcam, Cambridge, UK). The analyses were performed using a SUNRISE ELISA reader (Tecan, Salzburg, Austria) at 450 nm. A panel of cytokines, chemokines, and growth factors (FGF, eotaxin, G-CSF, GM-CSF, IFN-γ, IL-1β, IL-1ra, IL-2, IL-4, IL-5, IL-6, IL-7, IL-8, IL-9, IL-10, IL-12 (p70), IL-13, IL-15, IL-17a, IP-10, MCP-1, MIP-1α, MIP-1β, PDGF-BB, RANTES, TNF-α, VEGF) was quantified using commercially available Bio-Plex Pro TM human Cytokine 27-plex Assay (BIO-RAD, California, USA) according to the manufacturer’s protocol. The analysis was performed using the luminex BIO-PLEX 200 System (Bio-Rad, California, USA).

### Immunohistochemistry

Samples of synovial tissue were obtained from five patients with RA (5 females, with a mean (SD) age of 67 (7) years) from one knee joint, two elbow joints, and two small hand joints. The control group comprised of synovial tissue from four patients with OA (2 females and 2 males, with a mean (SD) age of 63 (8) years) from three knee joints and one hip joint. All synovial tissue samples were obtained during joint surgery, embedded in paraffin, and cut into 5-μm-thick sections. Subsequently, the sections were deparaffinised and rehydrated. Endogenous peroxidase activity was inhibited by adding DAKO Dual Endogenous Enzyme Block (Agilent, Santa Clara, CA, USA) for 10 min, and non-specific hydrophobic binding activity was prevented by adding 2% bovine serum albumin (BSA, Thermo Fisher Scientific, Waltham, Massachusetts, USA) diluted in PBS. In order to detect IL-40 in the synovial tissue, the slides were immunoprobed with primary rabbit polyclonal C17orf99 antibody (Sinobiological, Eschborn, Germany) diluted 1:500 in 2% BSA in PBS, and were incubated overnight at 4°C. Isotype IgG Universal Negative Control for IS-Series Rabbit Primary Antibodies (Agilent, Santa Clara, CA, USA) was used as a negative control. After rinsing with PBS buffer, HRP conjugated polyclonal goat anti-rabbit secondary antibody (Agilent, Santa Clara, CA, USA) diluted 1:200 in 2% BSA in PBS was added for 1h at room temperature (RT). Liquid DAB + Substrate (Dako Cytomation, Glostrup, Denmark) was used as a chromogen. The slides were counterstained with Mayer’s Haematoxylin solution. Evaluation of results was performed using an Olympus BX53 microscope with DP80 Digital Microscope Camera and CellSens Standard V1.18imaging software (Olympus, Philadelphia, PA, USA).

### Immunofluorescence

Synovial tissue samples from patients with RA (3 females and 1 male, with a mean (SD) age of 62 (16) years) were obtained and processed as described above. At first, the 5-μm-thick slides were immunoprobed with rabbit polyclonal C17orf99 antibody (Sinobiological, Eschborn, Germany) diluted 1:700 in 2% BSA in PBS overnight at 4°C. Then, goat anti-rabbit IgG H&L Alexa Fluor 488 (Abcam, Cambridge, UK) diluted 1:200 in 2% BSA in PBS was added as a secondary antibody for 1h at RT. Consequently, slides were incubated with primary antibodies against CD68 (1:100), CD3 (1:100), CD20 (1:50), CD1a (1:100) all from Abcam (Cambridge, UK) and against myeloperoxidase (MPO,1:200; Invitrogen, Carlsbad, CA, USA) overnight at 4°C. After incubation, goat anti-mouse IgM mu chain Alexa Fluor 647 (Abcam, Cambridge, UK) diluted 1:200 in 2% BSA in PBS was added for 1h at RT. DAPI (Thermo Fisher Scientific, Waltham, Massachusetts, USA) was used to stain the cell nuclei. Evaluation of results was performed using a BX53 microscope with DP80 Digital Microscope Camera and CellSens Standard 3.1 imaging software (Olympus, Philadelphia, PA, USA).

### Cell Cultures and *In Vitro* Experiments

RA synovial fibroblasts were obtained from biopsies from RA patients (n=9) as previously described (21), and were cultured in DMEM medium (Gibco-Thermo Fisher, Waltham, MA, USA) supplemented with 10% fetal bovine serum (FBS) at 37°C in a 5% CO_2_ humidified atmosphere. Cells between passages 4 and 8 were seeded on 6-well plates (1.0 × 10^5^ cells/well) and stimulated with IL-40 (C17ORF99 Recombinant Protein, N-terminal 10 x His-tagged and C-terminal Myc-tagged, produced in E.coli, low endotoxin, sterile filtered from Aviva Systems Biology, San Diego, CA, USA) at concentrations of 100, 200 and 250 ng/ml or with lipopolysaccharide (LPS, 100 ng/ml, LPS from E. coli O26:B6, Sigma-Aldrich, St. Louis, MO, USA) for 24 hours, cell culture supernatants were collected after 24 hours and stored at -80°C. The range of IL-40 concentrations used for *in vitro* experiments was selected based on physiological concentrations detected in the serum and in the synovial fluid of RA patients.

### Statistical Analysis

The data are presented as median and interquartile range (IQR) if not stated otherwise. Normality of data across analysed groups was tested by Kolmogorov-Smirnov test with 5% level of statistical significance. For analysis of differences between groups, Mann-Whitney U-test for non-paired data was performed. Wilcoxon matched-pairs signed rank test was applied for the *in vitro* data. Bivariate relationships between variables under study were determined by Spearman’s correlation coefficient. Multivariate regression analysis was used to predict levels of IL-40 in the serum and synovial fluid by a set of predictors. The predictors for both dependent variables (serum IL-40 and synovial fluid IL-40) were selected based on significant bivariate associations. Highly collinear predictors were excluded from the analysis, and only the best predictor (with the highest correlation with dependent variable) was retained in the regression model. Longitudinal observations of IL40 were examined using two-way (cohorts 2 and 3 by time) repeated-measures analysis of variance (RM-ANOVA) to assess the inter-group differences, followed by one-way RM-ANOVA with least significant difference (LSD) post-hoc tests conducted within each group. P values less than 0.05 were considered statistically significant (*p<0.05, **p<0.01, ***p<0.001, ****p<0.0001). GraphPad Prism 6 (GraphPad Software, La Jolla, CA, USA) and IBM SPSS 25.0^®^ (Chicago, IL, USA) were used to perform the analysis.

## Results

### Increased Expression of IL-40 in the RA Synovial Tissue

**Figure 1:**
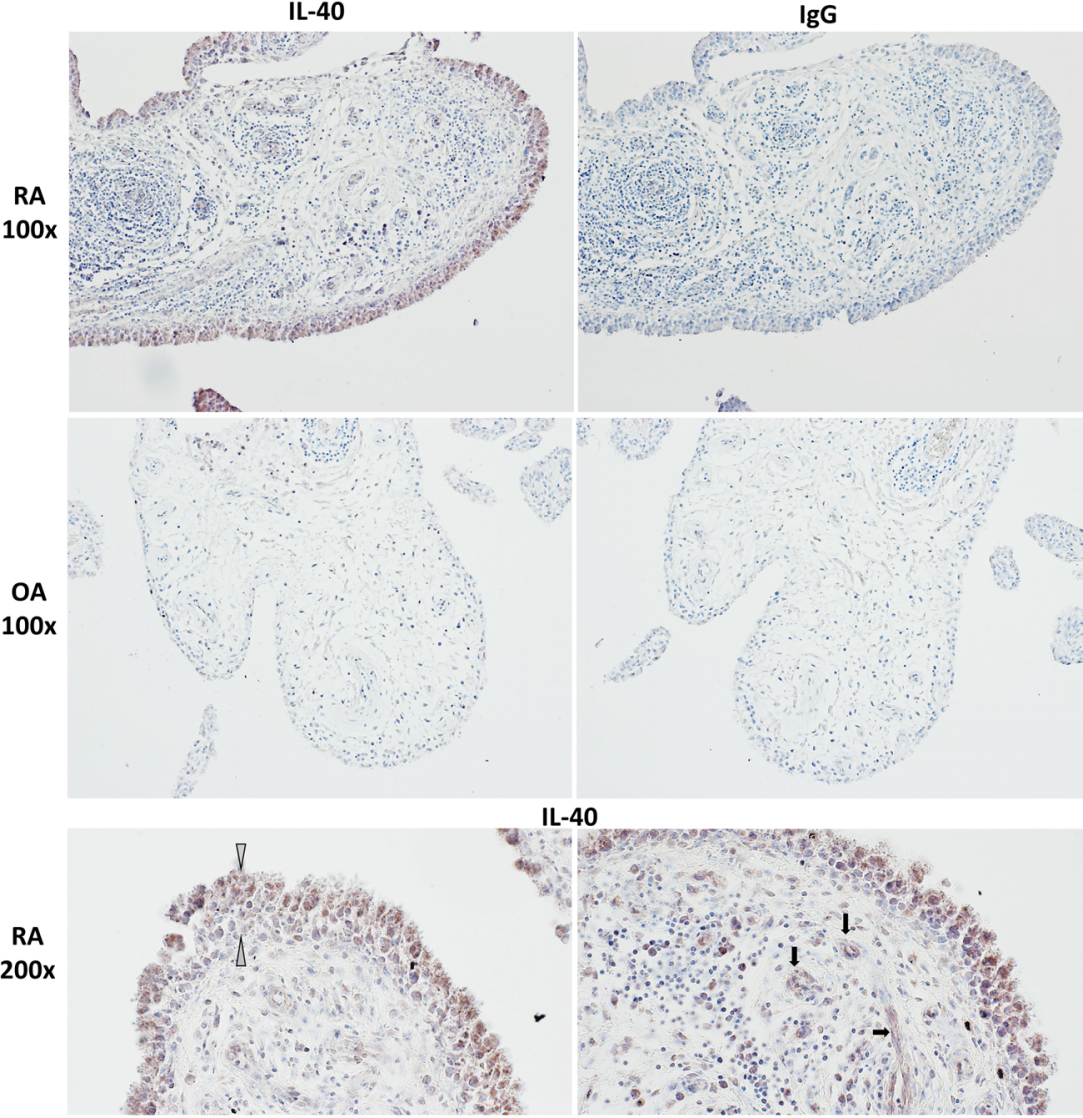
IL-40 in the synovial membrane of patients with rheumatoid arthritis (RA) and osteoarthritis (OA). Intensive IL-40 positivity was observed in RA, especially in the hyperplastic lining layer and within the inflammatory infiltrates of the RA synovium. Moderate expression of IL-40 was detected in the endothelial cells. IL-40 expression was quite sparse with only few IL-40 positive immune cells within the OA synovial tissue. Rabbit IgG was used as an isotype control. The white arrows point to the hyperplastic lining layer, and the black arrows point to capillaries. Representative images of immunohistochemistry staining are shown at × 100 magnification, detailed view at × 200 magnification (RA, n = 5; OA, n = 4).

**Figure 2:**
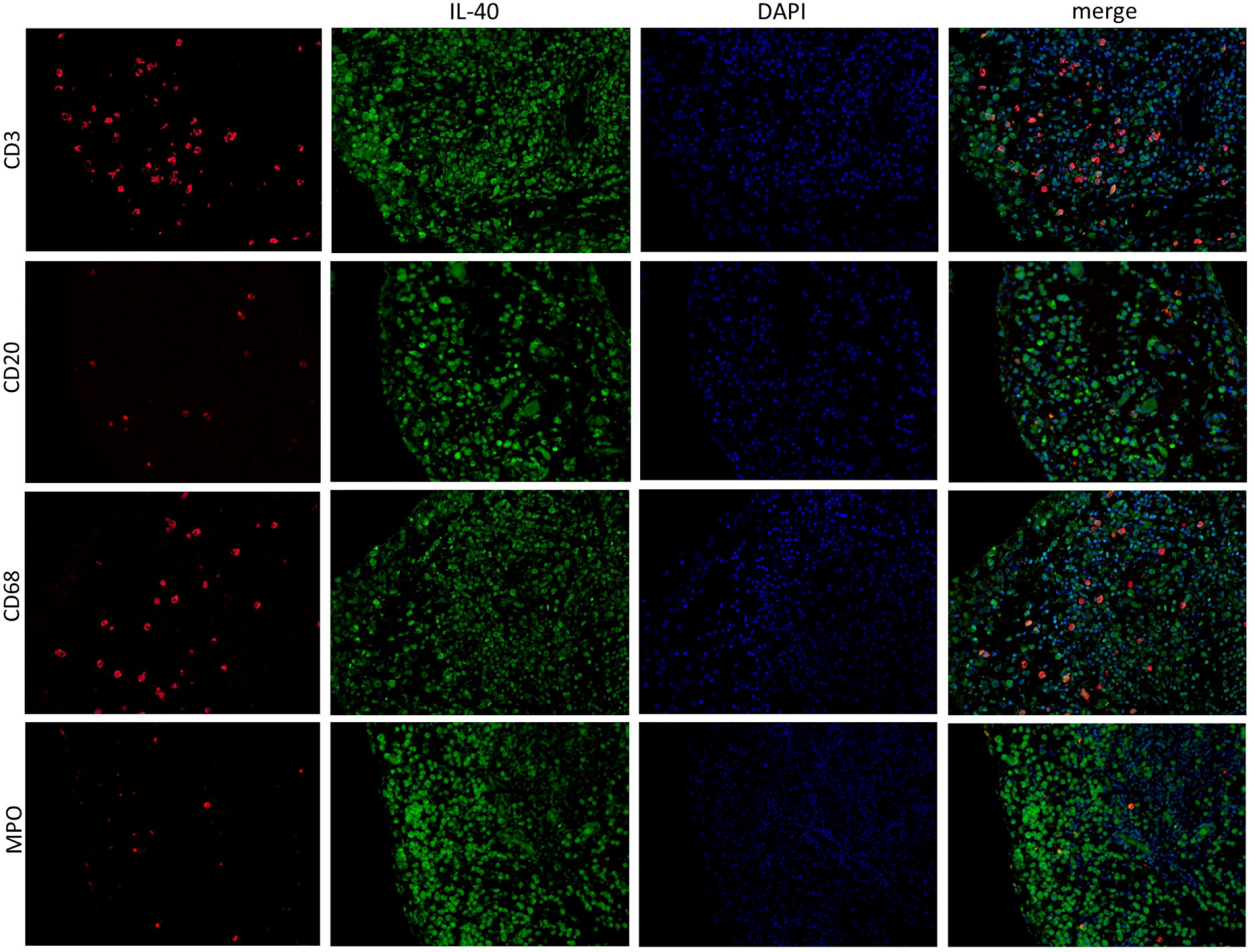
IL-40 is expressed by immune cells in the synovial membrane of patients with rheumatoid arthritis (RA). In addition to the synovial lining, IL-40 positivity (green) was observed in infiltrating cells of RA synovial tissue, demonstrated by specific marker staining (red) for T lymphocytes (CD3), B lymphocytes (CD20), macrophages (CD68), and neutrophil myeloperoxidase (MPO). Nuclei were stained by DAPI (blue). Representative images of immunofluorescence staining are shown at × 200 magnification (RA, n = 4). DAPI, 4’,6-diamidino-2-phenylindole.

IL-40 was detected in both RA and OA synovial tissue (**Figure 1**). The IL-40 expression was significantly enhanced in RA compared to OA synovial tissue, particularly within the inflammatory infiltrate (**Figure 1**). Moreover, the hyperplastic lining layer of the RA synovial tissue showed significant positivity for IL-40. Moderate expression of IL-40 was detected in the endothelial cells in RA synovium. In contrast, OA synovium had negligible IL-40 expression with only sparse staining positivity within the tissue (**Figure 1**). Further analysis of RA synovium using double-immunofluorescence staining demonstrated co-localization of IL-40 with macrophages (CD68), B cells (CD20), T cells (CD3), and neutrophils (MPO) (**Figure 2**).

### Levels of IL-40 Are Up-Regulated in the Synovial Fluid and Serum of RA Patients (Cohort 1)

**Figure 3:**
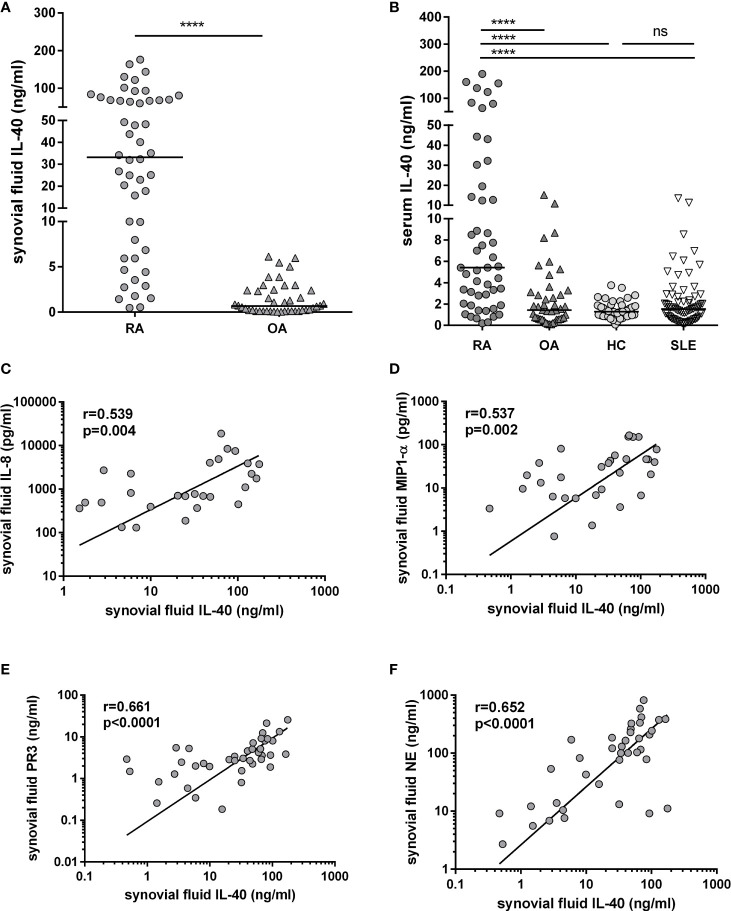
Up-regulation of IL-40 in patients with rheumatoid arthritis (RA) and its association with the levels of chemokines and markers of NETosis. Levels of IL-40 in synovial fluid are significantly higher in RA patients compared to osteoarthritis (OA) **(A)**. Levels of IL-40 in serum are elevated in RA patients compared to OA patients, healthy controls (HC), and patients with systemic lupus erythematosus (SLE) **(B)**. Synovial fluid IL-40 significantly correlated with the levels of chemokines IL-8 **(C)** and MIP1-α **(D)** and with the markers of NETosis such as proteinase 3 (PR3) and neutrophil elastase NE **(E, F)** in the synovial fluid of patients with RA. ****p < 0.0001; ns, nonsignificant. The horizontal line represents the median. The association of IL-40 with chemokines was analysed using Spearman correlation.

IL-40 is significantly up-regulated in the synovial fluid of RA in contrast to OA patients [33.2 (6.6-68.9) vs. 0.7 (0.1-2.4) ng/ml; p<0.0001] (**Figure 3A**). Also, serum IL-40 is increased in RA patients compared to OA, HC or SLE [5.4 (12.0-22.2) vs. 1.4 (0.6-3.1), 1.4 (1.0-1.9) or 1.5 (0.7-2.7) ng/ml, respectively; p<0.0001 for all], (**Figure 3B**). Interestingly, IL-40 is markedly enhanced at local sites of inflammation in the RA synovial fluid compared to RA serum [33.2 (6.6-68.9) vs. 5.4 (2.0-22.2) ng/ml; p<0.0001]. The opposite phenomenon is present in OA, where systemic levels of IL-40 are higher compared to the synovial fluid IL-40 [1.4 (1.0-1.9) vs. 0.7 (0.1-2.4) ng/ml; p<0.0001]. Levels of IL-40 in the serum or the synovial fluid were not affected by sex or treatment with conventional synthetic disease-modifying antirheumatic drugs (csDMARDs) or glucocorticoids (GCs) (**Supplementary File 1**). No association of IL-40 levels with age was found. Moreover, the levels of IL-40 are markedly up-regulated in double-positive (RF+/anti-CCP+) compared to double-negative (RF-/anti-CCP-) patients with RA in the synovial fluid [66.6 (41.5-93.1) vs. 9.9 (3.7-24.0) ng/ml; p<0.0001], as well as in the serum [10.9 (4.2-96.0) vs. 3.3 (1.6-6.6) ng/ml; p<0.001]. Additional data regarding the relation of IL-40 levels to the autoantibody positivity are summarized in **Supplementary File 2**.

### IL-40 Is Associated With RA Disease Activity and Levels of Autoantibodies (Cohort 1)

There is a significant association between the levels of IL-40 in serum and synovial fluid in both RA and OA patients (r=0.705, p<0.0001 and r=0.791, p<0.0001, respectively). Levels of IL-40 in the synovial fluid of RA patients positively correlated with ESR (r=0.440, p=0.003), disease activity DAS28-ESR (r=0.346, p=0.025) and synovial fluid leukocyte count (r=0.443, p=0.002). A weak correlation was observed between synovial fluid IL-40 and the number of swollen joints (r=0.284, p=0.049). Serum IL-40 was weakly associated to ESR (r=0.318, p=0.038) but not to DAS28-ESR (r=0.194, p=0.218) in RA patients.

Levels of IL-40 significantly correlated with the levels of anti-CCP and RF-IgM autoantibodies in the serum (r=0.423, p<0.01 and r=0.631, p<0.0001, respectively) and in the synovial fluid (r=0.548, p<0.001 and r=0.684, p<0.0001, respectively). Bivariate associations of serum and synovial fluid IL-40 with major clinical and laboratory parameters are summarized in **Table 2**. All the selected parameters (IgM, anti-CCP, and ESR) remained significantly associated with IL-40 in the synovial fluid, while only IgM and anti-CCP with IL-40 in the serum in a multivariate regression model. IgM had the strongest association with IL-40 in both synovial fluid and serum (standardized β = 0.45 and 0.38, respectively), even after adjusting for other variables in the model (**Table 3**).

**Table 2:** Bivariate correlations of IL-40 levels with clinical and laboratory parameters in patients with RA (cohort 1).

		Serum	Synovial fluid
**Laboratory parameters**	**CRP**	0.021	0.190
	**ESR**	0.318*	0.440**
	**RF-IgM**	0.631****	0.684****
	**anti-CCP**	0.423**	0.548***
	**SF- leukocyte count**	0.089	0.443**
**Clinical parameters**	**DAS28 (ESR)**	0.194	0.346*
	**SJC**	0.106	0.284*
	**TJC**	0.009	-0.016

Data were analysed using Spearman correlation and are presented as correlation coefficient r. *p < 0.05, **p < 0.01, ***p < 0.001, ****p < 0.0001. anti-CCP, anti-cyclic citrullinated peptide antibody; CRP, C-reactive protein; DAS, disease activity score; ESR, erythrocyte sedimentation rate; IgM, immunoglobulin M; RF, rheumatoid factor; SF, synovial fluid; SJC, swollen joint count; TJC, tender joint count.

**Table 3:** Multivariate regression analysis predicting IL-40 levels in the serum and synovial fluid of RA patients based on laboratory parameters (cohort 1).

Variables	Serum		Synovial fluid	
	β (95% CI)	P-value	β (95% CI)	P-value
IgM	0.376 (0.026-0.184)	**0.011**	0.450 (0.057-0.183)	**0.0001**
anti-CCP	0.332 (0.009-0.076)	**0.014**	0.302 (0.011-0.064)	**0.007**
ESR	-0.147 (-0.895-0.265)	0.280	0.234 (0.021-0.941)	**0.041**

Dependent variable: IL-40; β-standardized regression coefficient; CI, confidence interval.

anti-CCP, anti-cyclic citrullinated peptide antibody; ESR, erythrocyte sedimentation rate; IgM, immunoglobulin M. Statistically significant differences (p<0.05) are marked in bold.

### Synovial Fluid IL-40 Correlates With the Count and Activation of Neutrophils and With Markers of NETosis (Cohort 1)

In patients with RA, synovial fluid IL-40 significantly correlates with the number of synovial fluid neutrophils (r=0.375, p=0.017) and with the synovial fluid levels of chemokines IL-8 (r=0.539, p=0.004) and MIP-1α (r=0.537, p=0.002), (**Figures 3C, D**). Given the association of IL-40 with the neutrophil attractant and activator IL-8, we sought to analyse the relation between IL-40 and markers of NETosis. We found that synovial fluid IL-40 strongly correlates with the levels of proteinase 3 (PR3) (r=0.661, p<0.0001) and neutrophil elastase (NE) (r=0.652, p<0.0001) (**Figures 3E, F**). No relation was found between serum IL-40 and any of the studied cytokines, chemokines or NETosis markers (data now shown).

### B Cell Depletion Therapy, but Not TNF Inhibition, Significantly Reduced the Serum Levels of IL-40 in RA Patients (Cohorts 2 and 3)

**Figure 4:**
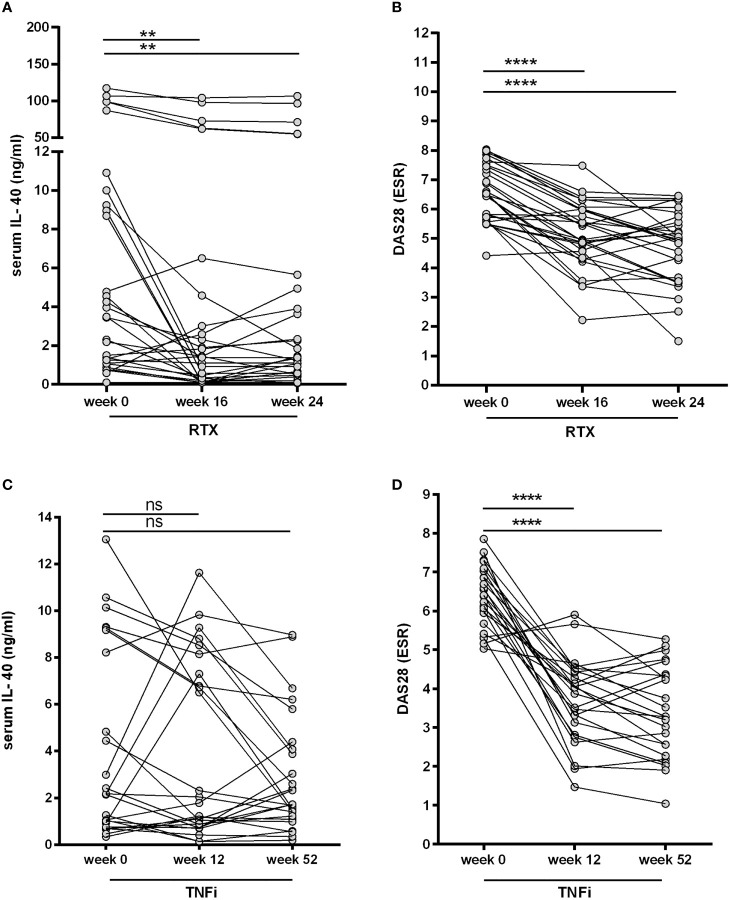
Effect of biological therapies on the serum IL-40 in patients with rheumatoid arthritis (RA). A decrease in IL-40 serum levels **(A)** was observed after the first series of rituximab (RTX) at weeks 16 and 24 and was associated with a drop in the disease activity (DAS28) **(B)**. No significant changes of IL-40 serum levels following therapy with TNF inhibitors (TNFi) after 12 and 52 weeks were detected **(C)**, even though disease activity (DAS28) markedly declined **(D)**. **p < 0.01; ****p < 0.0001; ns, nonsignificant.

To assess the role of B cell homeostasis on IL-40 production, we measured the serum levels of IL-40 prior to and after rituximab administration in patients with RA (cohort 2). Levels of circulating IL-40 significantly decreased in the majority of patients after 16 weeks [3.5 (1.2-9.6) vs. 1.4 (0.1-3.8) ng/ml, p = 0.003] and 24 weeks [3.5 (1.2-9.6) vs. 1.4 (0.5-4.4) ng/ml, p = 0.007] following rituximab treatment (**Figure 4A**). In parallel with that, significant depletion of peripheral B cells was achieved after 16 weeks [0.1 (0.1-0.3) vs. 0.001 (0.0-0.006) G/l, p < 0.0001] and 24 weeks of the therapy [0.1 (0.1-0.3) vs. 0.004 (0.001-0.03) G/l, p < 0.0001]. Rituximab treatment also resulted in a significant decrease in disease activity as represented by DAS28 (ESR) in week 16 [6.5 (5.7-7.5) vs. 5.0 (4.4-6.0), p < 0.0001] and week 24 [6.5 (5.7-7.5) vs. 5.0 (3.6-5.8), p < 0.0001], **Figure 4B**. No association was observed between the levels of IL-40 and B cell count at baseline or following the treatment (data not shown).

Furthermore, no significant changes in the serum levels of IL-40 were observed in cohort 3 treated with TNFi adalimumab after 12 weeks [2.2 (0.8-8.7) vs. 1.8 (0.8-7.7) ng/ml, p = ns] and 52 weeks [2.2 (0.8-8.7) vs. 1.7 (1.2-4.2) ng/ml, p = ns] of the therapy (**Figure 4C**). Nevertheless, disease activity [DAS28 (ESR)] was significantly reduced in week 12 [6.4 (5.7-7.0) vs. 4.0 (2.9-4.4), p < 0.0001] and week 52 [6.4 (5.7-7.0) vs. 3.3 (2.3-4.4), p < 0.0001] following adalimumab therapy (**Figure 4D**). The inter-group analysis confirmed a significant difference in the change of serum IL-40 levels between cohort 2 and cohort 3 over the studied period of time (p = 0.004).

### IL-40 Stimulates the Expression of Pro-Inflammatory Cytokines/Chemokines and MMP-13 in RA Synovial Fibroblasts

**Figure 5:**
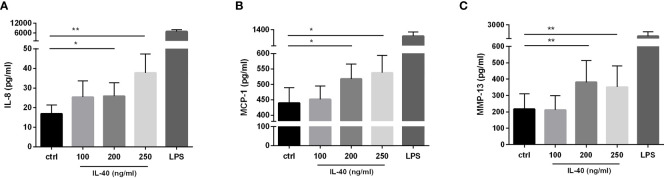
IL-40 dose-dependently induced the secretion of pro-inflammatory cytokines/chemokines and MMP-13 in RA synovial fibroblasts (n=9). RA synovial fibroblasts significantly up-regulate the secretion of interleukin (IL)-8 **(A)**, monocyte chemoattractant protein (MCP)-1 **(B)**, and matrix-metalloproteinase (MMP)-13 **(C)** when treated by IL-40 at concentrations of 200-250 ng/ml. Fibroblasts were stimulated for 24h prior to protein analysis. Data are represented as mean ± SEM. *p < 0.05, **p < 0.01 compared to unstimulated fibroblasts (ctrl).

RA synovial fibroblasts exposed to IL-40 up-regulated the secretion of IL-8 into the supernatants in a dose-dependent manner [250 ng/ml: 34.1 (11.8-62.7) pg/ml, p = 0.004; 200 ng/ml: 16.61 (9.9-51.8) pg/ml, p = 0.049; 100 ng/ml: 18.4 (7.1-41.7) pg/ml, p = ns, compared to the unstimulated control: 15.0 (2.6-24.9) pg/ml] (**Figure 5A**). Similarly, MCP-1 secretion by synovial fibroblasts significantly increased upon stimulation by IL-40 [250 ng/ml: 548.0 (368.6-686.1) pg/ml, p=0.042; 200 ng/ml: 486.5 (375.8-707.2) pg/ml, p = 0.016; 100 ng/ml: 425.8 (315.2-581.0) pg/ml, p = ns, compared to the unstimulated control: 402.7 (285.2-614.5) pg/ml] (**Figure 5B**). No significant changes in the levels of IL-6, RANTES or IFNγ were observed upon treatment with IL-40 (data not shown). The levels of the other cytokines/chemokines or growth factors included in the 27-plex assay were under the detection limit of the assay (the 27 analysed cytokines are listed in Methods).

In addition, we observed a significant effect of IL-40 on the secretion of metalloproteinase MMP-13 [250 ng/ml: 224.0 (40.5-637.5) pg/ml, p=0.008; 200 ng/ml: 223.0 (73.8-819.0) pg/ml, p = 0.008; 100 ng/ml: 79.0 (19.5-445.5) pg/ml, p = ns, compared to the unstimulated control: 89.0 (14.5-433.5) pg/ml] (**Figure 5C**). Stimulation of RA synovial fibroblasts by IL-40 did not affect the release of MMP-1 and MMP-3 (data not shown).

## Discussion

We are the first to demonstrate the implication of the new cytokine IL-40 in the pathogenesis of RA. We show local and systemic up-regulation of IL-40 in patients with RA and its association with disease activity, levels of autoantibodies, chemokines, and markers of NETosis. We also provide evidence that extracellular IL-40 enhances the secretion of chemokines and matrix-degrading enzyme MMP-13 in synovial fibroblasts.

IL-40 is a recently discovered B cell-associated cytokine related to immune response and B cell homeostasis (10, 17). Here we demonstrated that IL-40 is accumulated in the synovial tissue of RA patients. We observed intensive IL-40 staining within the hyperplastic synovial lining layer, indicating its expression by activated synovial fibroblasts. Also, immune cells infiltrating the RA synovial tissue tested positive for IL-40. According to Catalan-Dibene et al., IL-40 expression is associated with B cells (10); however, our data on synovial tissue indicate that besides B cells, other immune cells such as macrophages, T cells and neutrophils are involved in IL-40 expression in RA. This possibility is supported by our analysis of serum IL-40 in RA patients treated with B cell depleting agent rituximab. We showed that high levels of IL-40 decreased following rituximab therapy in 70% of patients, whereas the remaining 30% showed unchanged or even increased levels of serum IL-40. Indeed, no significant association of IL-40 levels with the number of B cell at baseline or following the therapy was found. Since the B cell depletion was accompanied by a decline in disease activity, dysregulation of other immune cells might have occurred and contributed to the changes in the IL-40 levels following the treatment. Altogether, these data suggest that IL-40 is produced by multiple cell types in RA. Of note, serum levels of IL-40 do not seem to be significantly affected by the treatment with TNF inhibitor adalimumab. Targeting specific upstream events, such a B cells activation, rather than TNF itself, may be responsible for reducing IL-40, which indicates that IL-40 may be related to specific mechanisms in the pathogenesis of RA linked to B cells and their regulatory effects.

Furthermore, IL-40 was elevated in the paired samples of serum and synovial fluid of RA patients compared to OA. In addition to that, we assessed IL-40 in the serum of patients with SLE, a systemic autoimmune rheumatic disease characterised by abnormalities and hyperactivity of B cells (22). Surprisingly, the serum levels of IL-40 in SLE were comparable to the ones in OA and healthy controls, suggesting no specific association of IL-40 with neither B cells nor the systemic nature of the disease. These findings imply that the enhanced systemic levels of IL-40 in RA may be related to the local inflammation and originate from the inflamed joints rather than from systemic inflammation. In support of this claim, levels of IL-40 in the RA synovial fluid are about six times higher than those in the serum and are strongly associated with each other. In addition, IL-40 correlates with several indicators of local inflammation such as synovial fluid leukocyte count, but also with the disease activity score, or the number of swollen joints, but not with CRP. In light of these findings, it should also be stressed that only a negligible amount of IL-40 was detected in the OA synovial fluid, and the levels of IL-40 in the OA serum were comparable to the ones found in healthy individuals. This observation further reinforces the hypothesis that IL-40 diffuses from the RA joints to the blood circulation and reflects the intensity of the local inflammation.

We also demonstrated that IL-40 is significantly associated with the levels of RF-IgM and anti-CCP autoantibodies and thereby with the activation of B cells, which is in agreement with previously reported data (10). Of particular interest is the correlation of IL-40 with anti-CCP, a finding which outlines a possible implication of IL-40 in the process of citrullination and autoimmune reaction in RA. It is well established that the anti-CCP production in RA is fuelled by citrullinated antigens released during the formation of neutrophil extracellular traps - NETosis (23) and that anti-CCP antibodies play an important role in the early phases of RA development (24). In this regard, we showed that synovial fluid IL-40 correlates with the number of neutrophils in the synovial fluid and with the synovial fluid levels of IL-8, a neutrophil chemoattractant and potent inducer of NETosis (25). Consistently, IL-40 co-localized with MPO, one of common neutrophil markers, in the RA synovial tissue. In addition, the strong correlation of synovial fluid IL-40 with the levels of proteinase 3 and neutrophil elastase further highlight the link of IL-40 to neutrophils and NETosis. Moreover, our unpublished preliminary data indicate that IL-40 is released by RA neutrophils undergoing NETosis. A question which therefore arises is whether the high concentration of IL-40 in the synovial fluid may be a result of the crosstalk between the activated B cells and neutrophils in RA joint. In addition to all that, *in vitro* data show that RA synovial fibroblasts enhanced the IL-8 secretion upon IL-40 stimulation. Given the abundance of IL-40 in the RA synovial fluid, we can speculate that IL-40 stimulates fibroblasts in the synovial lining layer to produce IL-8, which in turn, can activate synovial fluid neutrophils and thereby fuel the autoimmune reaction in RA joints. Moreover, IL-40 up-regulates the secretion of pro-inflammatory chemokine MCP-1 by synovial fibroblasts. MCP-1 has recently been demonstrated to act on synovial fibroblasts to promote their aggressive phenotype (26). Thus, it is tempting to assume that IL-40 may be implicated in the development of synovial hyperplasia. Noteworthy is the association of synovial fluid IL-40 with the levels of MIP-1α, which not only functions as multifunctional chemokine and neutrophil attractant (27) but, according to Jordan et al., also represents a potent regulator of bone resorption in arthritis (28). In the context of bone remodelling, the correlation of IL-40 with anti-CCP and IL-8 should be emphasized, as IL-8 is involved in the anti-CCP-driven osteoclast activation and bone loss (29, 30). Last but not least, we revealed that IL-40 acts on the RA synovial fibroblasts to up-regulate the secretion of MMP-13, a key molecule in the cartilage degradation network (31). These data imply that IL-40 may play a particular role in the RA joint damage, a subject that requires further investigation.

Some limitations of the study should be taken into account. The relationship between the IL-40 expression in the synovial tissue and fluid could not be assessed since the tissue samples were obtained from a group of patients different from those who underwent synovial fluid aspiration. Furthermore, the correlation between IL-40 and increased NETosis observed in the synovial fluid could not be further explored in patients following biological therapies, as there are no synovial fluid samples available after the treatment.

Notwithstanding these limitations, this study shows for the first time an implication of IL-40 in the pathogenesis of RA. In fact, these novel findings may pave the way for placing IL-40 into the portfolio of molecules involved in the immune reaction and tissue remodelling control in RA. Also, this work may inspire further studies on IL-40 in other autoimmune diseases.

## Conclusion

Taken together, our results show that IL-40 is elevated in RA and decreases following the B cell depleting therapy. Moreover, IL-40 correlates with disease activity, autoantibodies, chemokines, and markers of NETosis, indicating its potential implication in RA development. In addition, IL-40 up-regulates chemokines and MMP-13 in synovial fibroblasts, which implies its potential role in inflammation and tissue destruction in RA.

## Data Availability Statement

The raw data supporting the conclusions of this article will be made available by the authors, without undue reservation.

## Ethics Statement

The studies involving human participants were reviewed and approved by Institutional Ethics Committee of Institute of Rheumatology, Prague, Czech Republic. The patients/participants provided their written informed consent to participate in this study.

## Author Contributions

LAC and LŠ were responsible for the study concept and design. LŠ, MT, DT, MO, and JZ recruited the patients and collected the clinical data. HH, VB, and AN carried out the ELISAs and analysed the data. DV performed the biopsies of synovial tissues. VB, AN, and LAC performed the immunohistochemistry and immunofluorescence experiments. AN and LAC performed the *in vitro* experiments. MK, MT, and LAC carried out the statistical analysis. LAC, AN, and LŠ were responsible for data interpretation and manuscript preparation. JV, LŠ, MT and KP revised the manuscript and gave their final approval of the version to be published. All authors contributed to the article and approved the submitted version.

## Funding

This study was funded by Research Project AZV-NU21-05-00276 of the Agency for Healthcare Research of the Czech Republic and by Research Project No. 023728 of the Ministry of Health of the Czech Republic, Charles University project SVV 260 523, and BBMRI-CZ LM2018125.

## Conflict of Interest

The authors declare that the research was conducted in the absence of any commercial or financial relationships that could be construed as a potential conflict of interest.

## Publisher’s Note

All claims expressed in this article are solely those of the authors and do not necessarily represent those of their affiliated organizations, or those of the publisher, the editors and the reviewers. Any product that may be evaluated in this article, or claim that may be made by its manufacturer, is not guaranteed or endorsed by the publisher.

## Abbreviations

anti-CCP, Anti-cyclic citrullinated peptides; BSA, Bovine serum albumin; bDMARDs; biological disease-modifying antirheumatic drugs; c17ORF99, Chromosome 17 open reading frame 99; CRP, C-reactive protein; csDMARDS, Conventional synthetic disease-modifying antirheumatic drugs; ctrl, Unstimulated cells; DAPI, 4’,6-diamidin-2-fenylindol; DAS28, Disease activity score in 28 joints using the erythrocyte sedimentation rate; DMEM, Dulbecco’s Modified Eagle Medium; ELISA, Enzyme-linked immunosorbent assay; ESR, Erythrocyte sedimentation rate; FBS, Fetal bovine serum; FGF, Fibroblast growth factors; GCs, Glucocorticoids; G-CSF, Granulocyte colony stimulating factor; GM-CSF, Granulocyte-macrophage colony-stimulating factor; HC, Healthy control; IFN-γ, Interferon gamma; Ig, Immunoglobulin; IL, Interleukin; IP-10, Interferon gamma-​induced protein 10; IQR, Interquartile range; LPS, Lipopolysaccharide; MCP-1, Monocyte chemoattractant protein 1; MIP, Macrophage inflammatory protein; MMP, Metalloproteinase; MPO, Myeloperoxidase; NE, Neutrophil elastase; NETosis, process of externalization of neutrophil extracellular traps; NETs, Neutrophil extracellular traps; ns, non-significant; OA, Osteoarthritis; PDGF, Platelet Derived Growth Factor; PR3, Proteinase 3; RA, Rheumatoid arthritis; RANTES, Regulated on activation; normal T cell expressed and secreted; RF, Rheumatoid factor; RT, Room temperature; RTX, Rituximab; SD, Standard deviation; SJC, Swollen joint count; SLE, Systemic lupus erythematosus; TGF, Transforming growth factor; TJC, tender joint count; TNF, Tumor necrosis factor; TNFi, TNF inhibitors; VEGF, Vascular endothelial growth factor.
