# Supplementary material for: IL-40: A New B Cell-Associated Cytokine Up-Regulated in Rheumatoid Arthritis Decreases Following the Rituximab Therapy and Correlates With Disease Activity, Autoantibodies, and NETosis
Source: Front Immunol. 2021 Oct 21;12:745523. doi: 10.3389/fimmu.2021.745523 (PMC8566875; doi:10.3389/fimmu.2021.745523)
Supplement: Supplementary file 1 [file DataSheet_1.docx]

Supplementary Material


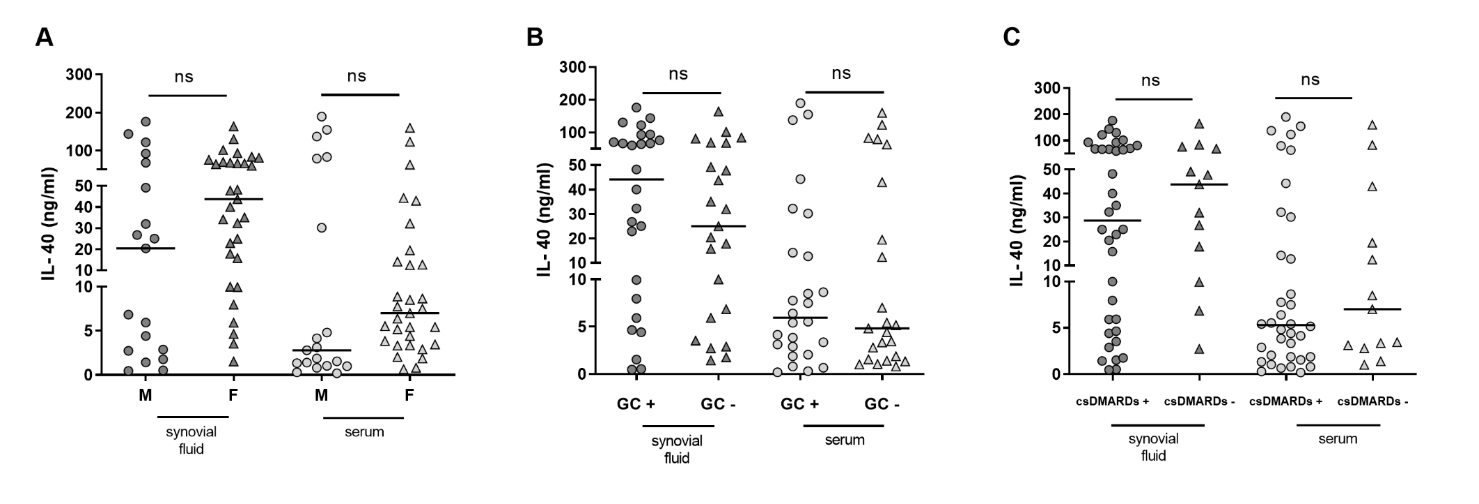


**Supplementary File 1**

Effect of conventional treatment and gender on the levels of IL-40 in patients with RA. The local and systemic levels of IL-40 are not affected by the gender of RA patients (A), treatment by glucocorticoids (GC) (B) or by conventional synthetic disease-modifying antirheumatic drugs (csDMARDs) (C). Data were alaysed by the Mann-Whitney U-test. The horizontal line represents median. ns, non significant

**Supplementary** **file 2**

Differences in the levels of IL-40 in the serum and synovial fluid of seropositive and seronegative patients with rheumatoid arthritis (RA). The data are presented as median (IQR). P values were determined by Mann-Whitney U-test. RF, rheumatoid factor (IgM); anti-CCP, anti-citrullinated protein antibody
